# Mechanical Tensions Regulate Gene Expression in the *Xenopus laevis* Axial Tissues

**DOI:** 10.3390/ijms25020870

**Published:** 2024-01-10

**Authors:** Fedor M. Eroshkin, Elena A. Fefelova, Denis V. Bredov, Eugeny E. Orlov, Nataliya M. Kolyupanova, Alexander M. Mazur, Alexey S. Sokolov, Nadezhda A. Zhigalova, Egor B. Prokhortchouk, Alexey M. Nesterenko, Andrey G. Zaraisky

**Affiliations:** 1 Shemyakin-Ovchinnikov Institute of Bioorganic Chemistry, Russian Academy of Sciences (IBCH RAS), 16/10 Miklukho-Maklaya Str., 117997 Moscow, Russia; 2 Laboratory of Development Biophysics, Department of Embryology, Faculty of Biology, Lomonosov Moscow State University, 119991 Moscow, Russia; 3 Federal State Institution “Federal Research Centre “Fundamentals of Biotechnology” of the Russian Academy of Sciences”, Leninsky Prospect, 33 Build. 2, 119071 Moscow, Russia; 4 Federal Center of Brain Research and Biotechnologies of Federal Medical-Biological Agency, 1 Build 10 Ostrovityanova Str., 117513 Moscow, Russia; 5 Department of Regenerative Medicine, Pirogov Russian National Research Medical University, 1 Build 70 Ostrovityanova Str., 117513 Moscow, Russia

**Keywords:** *Xenopus*, mechanical tension, mechanobiology, gene expression, *brachyury*, *goosecoid*, *otx2*, *six3*

## Abstract

During gastrulation and neurulation, the chordamesoderm and overlying neuroectoderm of vertebrate embryos converge under the control of a specific genetic programme to the dorsal midline, simultaneously extending along it. However, whether mechanical tensions resulting from these morphogenetic movements play a role in long-range feedback signaling that in turn regulates gene expression in the chordamesoderm and neuroectoderm is unclear. In the present work, by using a model of artificially stretched explants of *Xenopus* midgastrula embryos and full-transcriptome sequencing, we identified genes with altered expression in response to external mechanical stretching. Importantly, mechanically activated genes appeared to be expressed during normal development in the trunk, i.e., in the stretched region only. By contrast, genes inhibited by mechanical stretching were normally expressed in the anterior neuroectoderm, where mechanical stress is low. These results indicate that mechanical tensions may play the role of a long-range signaling factor that regulates patterning of the embryo, serving as a link coupling morphogenesis and cell differentiation.

## 1. Introduction

The idea that mechanical tensions (MTs) can regulate embryonic development has a long history [1,2]. It was also demonstrated by mathematical modelling that MTs are able, in principal, to play the role of a long-distance regulator of the embryonic patterning [3,4,5]. In support of this idea, evidence has emerged in recent years that experimentally demonstrates the role of MTs in signaling responsible for the regulation of gene expression within the embryo [6,7,8,9,10,11,12,13,14]. However, many questions in this field remain unaddressed. In particular, whether MTs play a role in gene regulatory signaling in a prominent morphogenetic process, such as the convergent extension of vertebrate axial structures, remains unknown. During this process, the chordamesoderm on the dorsal side of the embryo is extended during gastrulation and neurulation along the anteroposterior body axis because of active mediolateral intercalation of its cells towards the midline. Due to these cell movements, pushing forces are developed within the chordamesoderm, resulting in robust extension of the chordamesoderm and overlying neurectoderm along the anteroposterior axis and simultaneous shrinking in the perpendicular mediolateral direction.

In the present work, we performed experiments to identify genes with expression regulated by MTs in the stretched tissues of *Xenopus laevis* embryos. By combining explant stretching and full-transcriptome sequencing, we identified several genes with activated or inhibited expression in response to external mechanical stretching of the explant. Importantly, mechanically activated genes are expressed during normal development in the trunk, i.e., in the stretched part of the embryo. By contrast, genes inhibited by mechanical stretching are normally expressed in the anterior neuroectoderm, where mechanical stress is low. For the first time, to our knowledge, these results confirm that MTs may play a role in a long-range signaling factor that regulates global patterning of the embryo.

## 2. Results

### 2.1. The Head and Trunk Parts of the Neuroectoderm Experience Different Mechanical Tensions

As shown by tracing immediate mechanical reactions of the incised tissues, the head region of the neuroectoderm is compressed along the anteroposterior axis, whereas the trunk region of the neuroectoderm is stretched along this axis [15,16]. This finding indirectly suggests that if MTs do indeed play a role in regulatory signaling, they may specify different types of cell differentiation in the trunk and head regions of the neural anlage.

**Figure 1:**
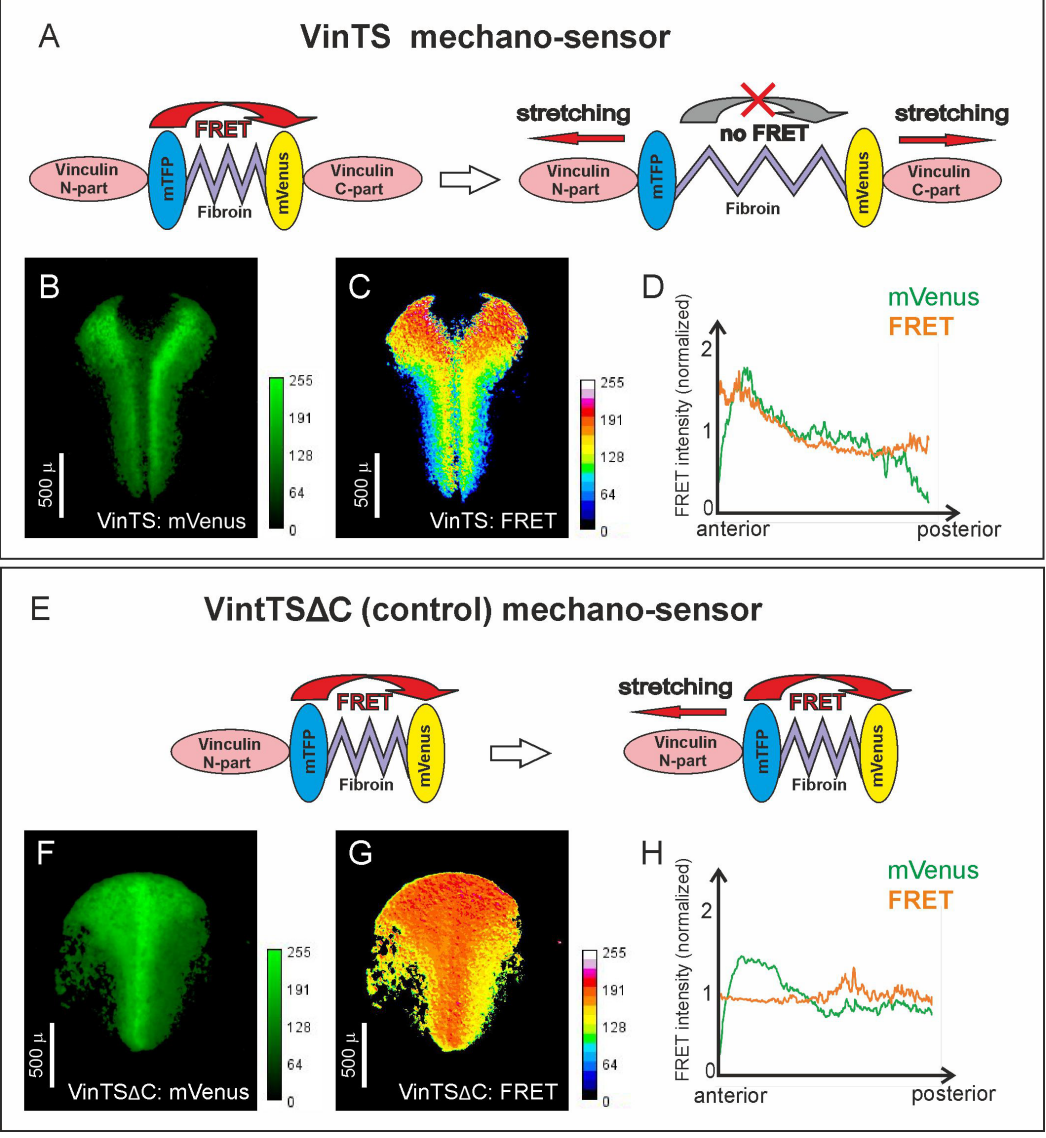
Genetically encoded mechanical sensor VinTS reveals different mechanical tensions in the head and trunk parts of the neuroectoderm. (**A**–**D**) VinTS mechanosensor. (**E**–**H**) VinTSΔC control. (**A**,**E**) Schema of sensor. (**B**,**F**) Images of the *Xenopus* embryo, microinjected with the VinTS mRNA, in the green channel. (**C**,**G**) The result of computer processing of images in green and cyan channels. Red color corresponds to low degree of MTs (high FRET); yellow and blue color corresponds to high degree of MTs (low FRET). (**D**,**H**) Vertical plot showing the distribution of signal intensity (green) and FRET (orange) along the anterior–posterior axis of the embryo.

First, to confirm that cells in the trunk and head regions of the neuroectoderm indeed undergo different MTs by a noninvasive method, we used the genetically encoded mechanical sensor VinTS [17]. This sensor was generated on the base of the vinculin molecule; in the middle of this molecule, a frequency resonance energy transfer (FRET) pair of fluorescent proteins, mTFP and Venus, separated by a fibroin elastic bridge, was inserted. As is known, the head part of Vinculin is attached to cell contacts, while its tail part binds the actin filaments. As a result, changes in MTs on cell contacts lead to stretching of VinTS, which results in changes of FRET intensity [17] (Figure 1A). A similar approach has been usen independently of us by [18], but we used a significantly lower amount of RNA (approximately 100 times lower) to reduce possible error resulting from a low percentage of mechanosensor incorporation into the cytoskeleton.

VinTS expression was established by injections of VinTS mRNA into early embryos followed by selecting of embryos with uniform distribution of VinTS fluorescence within the neural anlage (Figure 1A). During neurulation, compared with cells in the trunk region, cells in the anterior region of the neural plate were characterized by a significantly lower level of MTs applied to VinTS (Figure 1A, Movie S1). At the same time, experiments with the control VinTSΔC sensor, which demonstrates constant, tension-independent high FRET level, revealed no visible difference between head and trunk areas (Figure 1B, Movie S2).

To validate the data obtained using the VinTS mechanosensor, we analyzed whether the intensity of expression and uneven distribution of the mechanosensor within the embryo affects FRET. Image analysis showed a low correlation coefficient between the glow intensity in the green and cyan channels (Figure S1A). When comparing the VinTS mechanosensor with the control VinTSΔC, the normalized FRET value turned out to be almost twice as high in the control (Figure S1B). A summary graph of FRET distribution along the anterior–posterior axis clearly demonstrated the presence of a gradient of FRET within the neuroectoderm, whereas in the case of VinTSΔC microinjections, no FRET gradient was observed (Figure 1D,H and Figure S1D).

This result is consistent with the putative role of MTs as a factor that may regulate the anteroposterior patterning of the neural plate.

**Figure 2:**
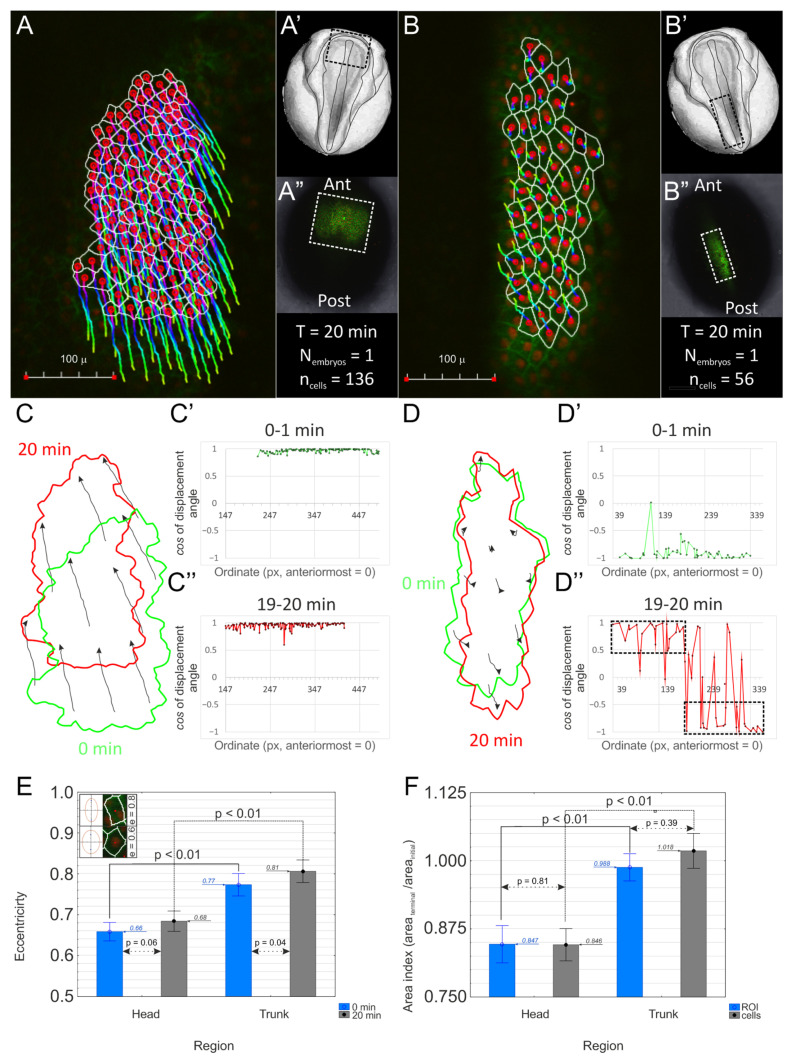
Morphometric analysis of tissue deformation. (**A**,**B**) To evaluate tissue deformation, we performed time-lapse imaging of cells in head (**A**) and trunk (**B**) regions of st. 14–15 *Xenopus* neurula during a 20 min interval. Insets in the right row show the position of segmented regions (enclosed with white dashed line) on scheme (**A′**,**B′**) and in imaged embryos (**A″**,**B″**) under lower magnification. Cell trajectories in (**A**,**B**) are color-coded: yellow–green indicates the initial part of the trajectory, blue—central, purple–red—terminal. (**C**,**D**) Tissue-level deformation evaluated by shape change of integral ROI including all segmented cells. Green and red counters reflect ROI shape at the first and last frame, respectively, of the time-lapse sequence. Individual trajectories of selected cells are visualized as black lines; arrows indicate direction of cell displacements inside ROIs. (**C′**,**D′**) and (**C″**,**D″**): To quantitatively describe spatial distribution of cell displacements underlying ROI deformation presented in (**C**,**D**), we calculated cosine of the angle between anteroposterior axis and direction of each cell displacement during two initial (**C′**,**D′**) and two terminal (**C″**,**D″**) frames in sequence and plotted this cosine value against ordinate (in pixels). We denote 0° (360°) displacement angle as shift towards anterior pole, and 180°—towards posterior. Thus, cosine values close to 1 indicate displacement towards the anterior pole, and values close to −1—towards the posterior pole. Note the marked translational shift of ROI in the head region without significant elongation on the contrary to the trunk region, that mostly remained in the field of view and was subjected to bidirectional ((**D″**) black dotted boxes) elongation. (**E**,**F**) To estimate cellular shape changes underlying observed ROI deformations, we analyzed distribution of cell eccentricities (**E**) for head (N_embryos_ = 1, n_cells_ = 136) and trunk (N_embryos_ = 1, n_cells_ = 56) regions at the start (blue) and the end (grey) of time-lapse series as well as area changes (**F**) of cells (blue) and small ROIs, including 10–11 cells (gray); columns show mean, whiskers—95% confidence interval. Inset in (**E**) (reprinted under Creative Commons 3.0 license) shows typical cell and respective ellipse shape for eccentricity = 0.6 and for eccentricity = 0.8. Note the slight (but statistically significant) increase in mean eccentricity in the trunk region while the apical cell area remains the same, reflecting cell elongation in the trunk region. At the same time, mean eccentricity in the head region did not change, while the apical cell area decreased, reflecting apical constriction.

To confirm morphogenetic relevance of reported changes in FRET intensity, we analyzed accompanying tissue deformation, as it may reflect mechanical status of the tissue [16,19,20]. For that purpose, we analyzed time-lapse images of cells in head (Figure 2A) and trunk (Figure 2B) regions of st. 14–15 *Xenopus* neurula collected during a 20 min interval. We found this time interval sufficient to detect core alteration in cell shapes (Figure S2). We first evaluated tissue-level deformation by analyzing shape change of integral ROIs including all segmented cells (Figure 2C,D). The minor axis of integral ROI was predominantly shortened both in head and trunk regions (12.04% and 13.62% respectively), while the length of the major axis did not change (head region, −0.17%) or was being elongated (trunk region, 13.43%). Cell displacements underlying tissue deformation were also qualitatively different in two regions. In the head region, directions of cells’ displacements were conserved along the entire anteroposterior axis and were headed toward the anterior pole (Figure 2C, black arrows), resulting in uniform spatial distribution of the order parameter (Figure 2C′,C″). This reflects translational shift of the entire region. On the contrary, in the trunk region, we detected spatially bifurcated cell displacements (Figure 2D, black arrows), and spatial distribution of the order parameter was noticeably separated in two successive domains (Figure 2D′,D″, black dotted boxes). At the same time, we found a statistically significant increase in the mean eccentricity of cells during observation only in the trunk region (*p* = 0.041), while the apical area of the vast majority of cells in this location did not change (Figure 2F). The latter indicates that the reported increase in eccentricity in the case of the trunk region is due to elongation of the cells. This elongation coincides with the predominant orientation of cells’ major axes parallel to the anteroposterior direction, i.e., the direction of presumed stretching (Figure S3D′,D‴).

In summary, these data lead us to a conclusion about the presence of tensile force in the neural plate that stretches the trunk region. To finally verify this suggestion, we compared morphometric data from intact embryos with data from double explants (Figure S3A,B) and found that stretched explants mostly correspond to the trunk region at 20 min (Figure S3D′–D‴). Although we found no evidence that the head region is being stretched in a similar way, we note that in the head region, lateral shrinkage together with persistent length of the major axis of the integral ROI is accompanied by nearly 16–17% reduction in the apical surface of cells (Figure 2F), indicating that anteroposterior shortening of the head region is mechanically constrained. Together with results obtained by the VinTS sensor, these data lead us to the conclusion that while the trunk region of the neural plate is stretched in the anteroposterior direction, stretching of the head region in this direction is being constrained, and the whole region is mostly characterized by shrinkage of the ROI area.

### 2.2. Modeling Stretching of the Trunk Neuroectoderm in Explants of the Dorsal Gastrula Ectoderm

**Figure 3:**
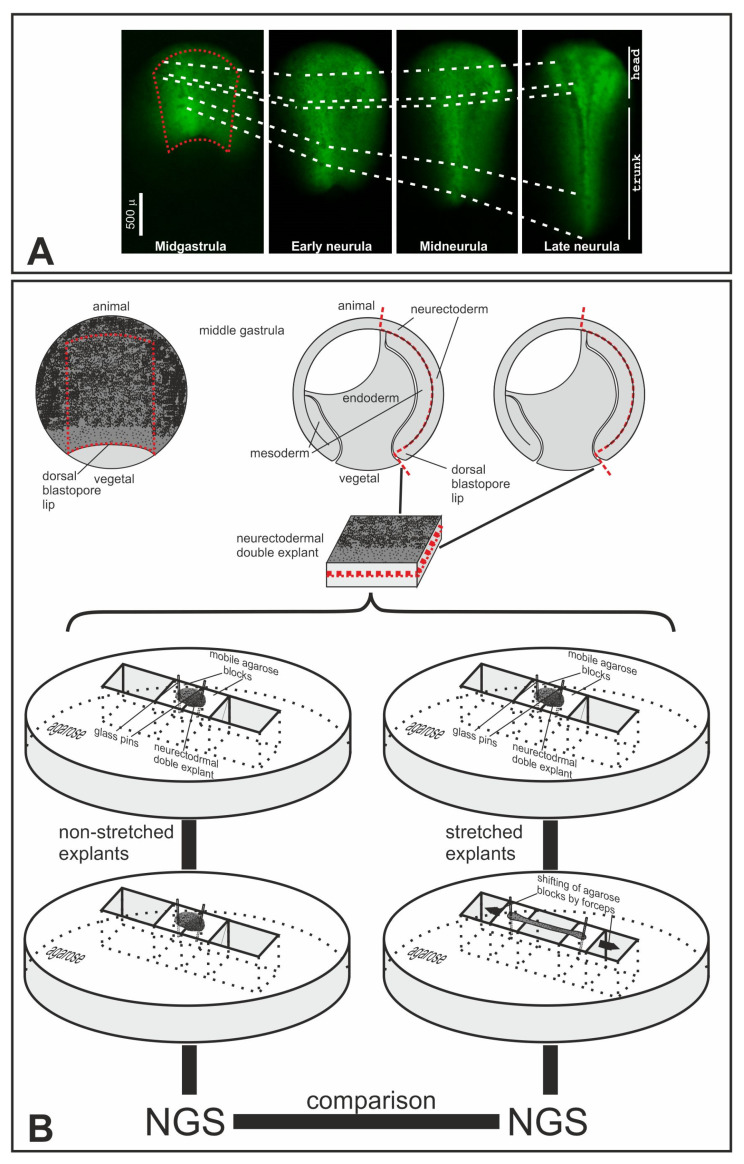
Convergent extension of the neurectoderm and schema of experiments in finding of genes regulated by mechanical tensions. (**A**) Photos of FLD-injected neural plate at different successive stages of development. White dashed lines connect at different stages the same noticeable features at the embryo surface. The red dashed line on the image of the midgastrula embryo indicates the approximate border of the neuroectodermal explants which had been excised at this stage. (**B**) Schema of experiments of explants stretching followed by NGS (see main text for details). Red dashed lines indicate the boundaries of the explants.

During the process of convergent extension, the chordamesoderm and overlying neurectoderm are extended several times along the anteroposterior body axis (Figure 3A) because of mediolateral intercalation of its cells towards the midline of embryo, generating MTs within cell sheets. Importantly, if the neuroectoderm is separated from the underlying chordamesoderm and isolated, it extends to a much lesser extent than it would in normal development [21]. Moreover, the outer layer apparently cannot extend by itself at all but is stretched in normal development only due to the tensile forces produced by underlying tissues [22]. These results indicate that the neuroectoderm is mainly stretched passively due to active elongation of the underlying chordamesoderm. This fact provides an opportunity to experimentally mimic the external stretching force that is normally generated by the extending chordamesoderm and identify mechanosensitive genes.

To this end, we cut off, at the middle gastrula stage, the rectangular explants, combined them in pairs, inner sides to each other, and used an earlier developed approach to stretch these sandwiches pinned to an agarose substrate by thin glass needles [23]. However, as the cells of the explants begin to actively rearrange in response to stretching, which results in relaxation of the explant after 10–15 min of stretching, this technique involves periodic changes in the needle positions. As a result, this leads to severe damage to the explants. In addition, standardizing the degree of stretching of explants is very difficult with this method.

**Figure 4:**
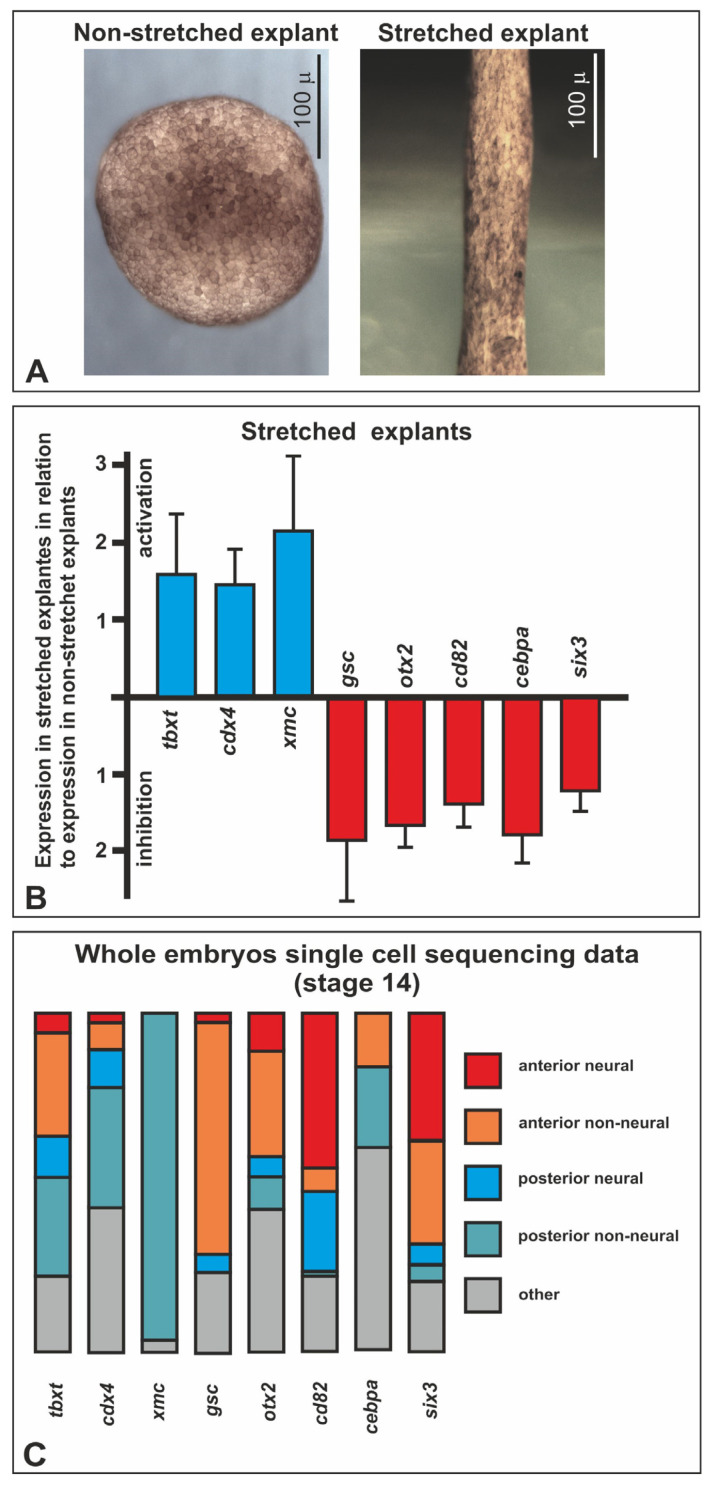
Analysis of the influence of the neuroectodermal explants stretching on gene expression. (**A**) Examples of explants nonstretched and stretched according to the schema shown in Figure 3B. (**B**) Genes whose expression changed in the explants upon the stretching, according to qRT-PCR data. Bars indicate the standard deviation. (**C**) Percentage expression distribution of selected MT-responsive genes in the indicated on the right groups of cells in midgastrula embryo (stage 14) according to the Jamboree single-cell sequencing database [24]. The group “anterior neural” includes such cell types as “anterior neural plate”; the group “anterior non-neural” includes such cell types as “anterior placodal area”, “cement gland primordium”, “eye primordium”, and “anterior neural crest”; the group “posterior neural” includes such cell types as “posterior neural plate”; the group “posterior non-neural” includes such cell types as “presomitic mesoderm”, “somite”, and “tail bud”; the group “other” includes other cell types (see *Xenopus* single-cell transcriptomic data analysis in Section 4.6 for details).

To circumvent these difficulties, we developed an original method that allowed us to stretch the explant by moving the agarose blocks to which it was pinned with glass needles instead of remounting the needles (Figure 3B and Figure S4). As a result, we could obtain reproducibly thoroughly elongated explants, with a final length up to 6–8 times the initial length. In the following experiments, the explants were stretched typically 2–3 times within 3 h, which approximately corresponds to the degree of stretching of the neuroectoderm in normal development. By using this technique, we obtained three independent series of pinned nonstretched (control) and stretched double-explants of the gastrula neuroectoderm (Figure 4A). It should be noted that no signs of self-stretching were observed in the control explants.

### 2.3. Differential Gene Expression in Stretched and Nonstretched Explants

To reveal possible differences between gene expression in stretched and nonstretched explants, we compared their transcriptomes using high-throughput sequencing (Table S2). Because this approach can produce large data scatter (since it is impossible to excise explants exactly the same), we verified the obtained data using quantitative RT-PCR. After such “filtering” of the top 84 genes (selected according to the LFC and P value criteria, Table S2), only 8 remained, the expression of which changed reproducibly in stretched explants compared to nonstretched ones. This set of genes included *cdx4*, *tbxt,* and *xmc*, which were upregulated, and *cd82*, *cebpa*, *gsc*, *otx2*, and *six3,* which were downregulated (Figure 4B). Interestingly, all three genes whose expression was upregulated in stretched explants (*cdx4*, *tbxt,* and *xmc*) are expressed in the trunk, stretched, region during normal development, whereas four of the five genes, whose expression was downregulated in stretched explants, are normally expressed in the head region of the embryo (*cd82*, *gsc*, *otx2*, and *six3*) (Figure 4C). It is important to note that all of these genes are involved in regulating the specification of the head and trunk parts of the anterior–posterior embryonic axis, respectively. Thus, the data obtained indicate the important role of MTs in this specification.

## 3. Discussion

One of the key problems in modern developmental biology is the identification of feedback between morphogenesis and gene expression. In this work, for the first time, we demonstrated the dependence of the expression of several genes that regulate the axial patterning along the anterior–posterior axis of the *Xenopus laevis* embryo on the distribution of MTs along this axis. MTs arise in the neuroectoderm and underlying axial mesoderm as a result of the dorsal convergence of cells in these tissues during gastrulation and neurulation. About 50 years ago, by measuring the dehiscence of small cuts margins in neuroectoderm at the neurula stage, the data indicated stronger MTs in its trunk region [16]. Here, we used two noninvasive methods (a genetically encoded FRET-based mechanosensor VinTS and cell morphometry) to evaluate the distribution of MTs. The fluorescent mechanosensor approach was also used, independently of us, by [18]. These authors used an actinin-based mechanosensor and obtained different, but not contrary to ours, results (the difference between neural and non-neural ectoderm), which can be explained by a different working principle of the sensor, as well as a significant difference in the levels of the sensors’ expression in the embryo (100 times higher than in our work, which can lead to a smaller percentage of its integration into the cytoskeleton).

To confirm the results obtained using VinTS sensor by an independent method, we analyzed the deformation of the tissue by means of the time-lapse cell morphometry. The overall length of a time-lapse sequence is two times shorter than the mean time of cell rearrangements in the neural plate outer ectoderm layer [25]. Thus, the reported deformations do not result from cell rearrangements and reflect mostly passive deformation of tissue. Our data led us to the conclusion that, in the trunk region at least, the outer ectoderm layer is being stretched during neurulation.

Further, we decided to identify the genes whose expression could be regulated by MTs arising in the stretched trunk part of the neurectoderm. The phenomenon of mechanodependent regulation of the expression of individual genes has been found in various biological models. In addition to the pioneering works [7,9], it has recently been shown that MTs, for example, play a key role in digit formation [12], gut rotation [13], and stem cell self-renewal [26]. However, to date, a full-scale search for mechanodependent genes in embryogenesis has not been carried out. Here, we conducted the first comprehensive search for mechanodependent genes using RNA-seq and found that at least three genes upregulated in the dorsal ectodermal midgastrula explants subjected to stretching (*tbxt*, *cdx4,* and *xmc*). Consistently, during normal development, these genes are expressed in the trunk, which is a more stretched region of the embryonic body axis. At the same time, the identified five stretch-inhibited genes (*gsc*, *otx2*, *cd82*, *six3,* and *cebpa*) are normally expressed in the less-stretched region, the head region of the embryo. Below, we briefly describe the data known from the literature on the expression and function of these eight genes in the early development.

### 3.1. Stretched-Activated Genes

*tbxt (brachyury)* is an important regulator of mesoderm specification as well as the development of the posterior region of the embryo [27,28]. During the middle gastrula stage, when we excised dorsal ectoderm explants for stretching experiments, this gene is expressed in two outer cell layers of the marginal zone, the region included in the explants [29]. Thus, the fact that our experiments revealed the expression of *tbxt*, the expression of which is predominantly associated with the embryonic mesoderm, cannot be considered unexpected. Interestingly, *tbxt/brachyury* was previously identified as mechanosensitive in *Danio* and *Nematostella* embryogenesis [7,30], which confirms the validity of our screen. Also, the increase in *tbxt* expression we observed may indicate the role of MTs in the regulation of the balance between neural and mesodermal differentiation in the neuromesodermal competent population. It is known that such a balance is determined by *sox2* and *tbxt* [31,32]. Respectively, MTs can guide neuromesodermal cells into mesodermal differentiation.

Similar to other *cdx* genes, *cdx4* is a well-known posterior regulator, which is expressed like *tbxt* in the outer layers of the gastrula marginal zone [33]. Knockdown of *cdx* genes results in severe posterior truncation, accompanied by a posterior shift and reduction in 5′ *Hox* gene expression. *cdx* and *wnt3* are components of a positive feedback loop operating at the posterior region of the embryo [34,35].

*xmc* is also expressed predominantly in the posterior part of the embryo, including outer layers of the gastrula marginal zone [36]. Knockdown of *xmc* results in the reduced elongation of the anteroposterior axis and inhibition of morphogenetic movements in embryos [36,37].

### 3.2. Stretched-Inhibited Genes

*gsc* is a well-studied key regulator of anterior structures. This gene promotes head organizer activity by repressing the canonical Wnt pathway in the anterior region of the embryo [38]. Knockdown of *gsc* leads to a reduction in head structures, including cyclopia and holoprosencephaly [39]. Moreover, *gsc* controls the anterior migration of cells [40].

*otx2* is another important regulator of head development [41,42]. *otx2* represses the expression of *tbxt* and *wnt11* and inhibits convergent extension movements [43].

*six3* also is a well-known regulator of anterior structures, such as telencephalon and eyes [44,45]. It is a marker of the anterior-most brain region not only in vertebrates, but in all studied bilaterian animals [46]. It functions in anterior neural plate specification, inhibiting Wnt and Bmp4 signaling [47,48,49,50,51].

*cebpa* encodes a homologue of vertebrate CCAAT/enhancer binding protein, C/EBP, that is poorly studied in *Xenopus*. This gene participates in the establishment and migration of the primitive myeloid lineage [52,53] and is involved in the regulation of the cell cycle as well as in the maintenance of body weight.

*cd82* is poorly studied in *Xenopus*. This gene is expressed in eyes and plays a role in endoderm patterning [54]. In humans, *cd82* is a marker of myogenic stem cells [55]. It regulates cell migration [56,57] and participates in bone growth [58]. According to our unpublished data, in early *Xenopus* embryos, *cd82* is expressed in the nervous system, predominantly in its anterior part.

### 3.3. Possible Mediators of the Mechanotransduction

During normal development, the longitudinally stretched, trunk part of the embryo is characterized by a higher levels of canonical Wnt/beta-Catenin signaling. Moreover, this signaling pathway reportedly mediates mechanodependent activation of *twist* in *Drosophila* and *notail/brachyury* in *Danio* upon beta-Catenin phosphorylation of tyrosine at position 667 [7,8].

It is logical to assume that in our case a similar mechanism is involved. However, of the genes identified in our screen, only *cdx4* is a transcriptional target of Wnt/beta-Catenin [59,60]. The role of Wnt/beta-Catenin in the regulation of the *xmc* gene is unknown. Moreover, *tbxt* has been shown not to be a target of Wnt/beta-catenin in *Xenopus* [61,62] unlike, for example, mouse ES cells [63]. Together with our unpublished data, this leads us to doubt the role of Wnt/beta-Catenin pathway as a mechanotransducer in our model system, and raises a logical question: What other mechanism(s) can mediate mechanotransduction?

A possible candidate for this role is the FGF/MAPK/Erk pathway. It is known from the literature that FGF/FGFR has a posteriorizing effect on *Xenopus* embryonic tissues [64,65,66,67]. This is in good agreement with our hypothesis about the role of mechanical stresses in the anteroposterior patterning of *Xenopus* embryos. In addition, all genes that are activated during explant elongation in our experiments (*cdx4*, *xmc,* and *tbxt*) are positively regulated by the FGF/MAPK cascade during normal development [36,68,69,70]. Recent papers describe the activation of FGFR and ERK kinases by mechanical stress (centrifugation and stretching) [71,72], which makes this cascade the most suitable candidate for the role of a mechanotransducer. Further research may shed light on this issue.

## 4. Materials and Methods

### 4.1. VinTS Cloning, RNA Microinjection, Image Acquisition, and Processing

Vinculin mechanosensor VinTS in a pcDNA3.1 plasmid [17] was obtained by the Addgene company (http://www.addgene.org/26019) (Watertown, MA, USA) (accessed on 15 May 2014). For in vitro synthesis of mRNA, the mechanosensor was recloned into the pCS2+ plasmid vector by HindIII (blunted)-XbaI restriction site (insert) and BamHI (blunted)-XbaI restriction site (vector). VinTSΔC control sensor was made by deletion of the NotI-NotI fragment, with subsequent subcloning into pCS2+ as was made for VinTS.

For mRNA synthesis, pCS2-VinTS and pCS2-VinTSΔC were linearized by Acc65I, and capped mRNA was synthesized using the SP6 mMESSAGE mMACHINE Transcription kit (Ambion, Naugatuck, CT, USA). Microinjections into *Xenopus* embryos were performed at the stage of two blastomeres (10 pg mRNA per each blastomere).

Time-lapse imaging of embryos was performed on a Leica M205 FA fluorescence microscope (Leica Camera AG, Wetzlar, Germany.) at the rate of 1 image pair every 2 min in CFP ET (excitation at 436/20 nm, emission at 480/40 nm) and GFP3 ET (excitation at 470/40 nm, emission at 525/50 nm) channels (excitation/emission peaks of the mTFP1.0 FRET donor were 462/492 nm and those of the mVenus FRET acceptor were 515/528 nm).

The series of images was processed using freely available ImageJ 1.46r software (https://imagej.net/ij/download.html, accessed on 10 April 2016). FRET intensity in each point of the embryo image was evaluated by the ratio of FRET donor and acceptor emission intensities using the integrated “image calculator” function. The ratio of intensities was visualized by the ImageJ 1.46r software using pseudocolors, with red corresponding to high level of FRET (low level of mechanical tensions) and yellow and blue corresponding to lower level of FRET (higher level of mechanical tensions).

### 4.2. FRET Validation

FRET measurements were performed as described with few modifications [73]: namely, background removal was performed by subtracting autofluorescence of 3–4 uninjected control embryos in the green and cyan channel. FRET gradients were calculated as follows: first, one-dimensional profiles of an integral FRET signal per line were generated along the anterior–posterior axis. Second, to obtain a mean FRET value per line, the FRET profile was divided by a binary mask profile, generated from the green channel. The resulting profile shows the mean FRET value along the axis, i.e., the FRET gradient of interest. Data were obtained in ImageJ 1.46r (see above) and processed in Excel 2016.

A possible influence of the injected mRNA distribution within the embryo on the FRET signal was checked by a correlation method: the green channel, showing the distributed injected material, was correlated with the cyan channel [74]. The obtained Pearson’s statistics were taken into account to weight the mRNA distribution influence on the FRET signal. Data were obtained in ImageJ using Image Correlator plugin. Data analysis was performed in Python using a customized code deposited under BSD-2 license (https://github.com/nataliyakolyupanova/vints_correlations/, accessed on 10 September 2023).

### 4.3. Cell Morphometry

To visualize cells for time-lapse imaging, we performed injection of membrane (GAP43-GFP) and nuclei (H2B-mCherry) RNA markers at 2 or 4 blastomere stage. Plasmids containing sequences of interest were kindly provided by Dietmar Gradl (Karlsruhe Institute of Technology, Karlsruhe). After injection (~230 pg in total volume 9.2 nL for each marker), embryos were cultured at until st. 14–15. For time-lapse imaging, embryos were transferred to 1× MMR, devitellinized with metal forceps, and, after 5 min, transferred to 35 mm glass-bottomed Petri dishes inside the well in LMP agarose on 1× MMR. Embryos were imaged using Olympus FV-10i LSCM equipped with 10× 0.4 NA UPLSAP objective under 2×–2.6× digital zoom and laser transmissivity = 50% for both channels. Images were collected every 1 min during 20 min intervals for both head and trunk regions.

Image segmentation was performed using the MATLAB module of EpiTools 2.1.6. image processing software (https://github.com/epitools/epitools-matlab/releases, accessed on 2 December 2016). Generated cell boundaries were exported in the form of *.png or *.TIFF images and further processed with EpiTools 2.1.6 plugins for the ICY bioimaging platform.

Exported data were further processed using the ICY bioimaging platform (https://icy.bioimageanalysis.org/, accessed on 10 September 2019), Microsoft Excel 2016, and STATISTICA 10 (http://statsoft.ru/resources/support/download.php?ysclid=lq3zdy6yv9567487005, accessed on 10 September 2019). The ICY bioimaging platform was used to extract coordinates of cell centroids, length of major and minor axes, and cell area values. Microsoft Excel 2016 was used to calculate cell displacement angles and corresponding cosine values based on coordinates of cell centroids, cell eccentricity based on length of major and minor axes, and area index based on cell area values. For detailed description of morphometric analysis, including plugins and formulas, see Supplementary Information. Statistical difference was calculated using the Mann–Whitney U-test in STATISTICA 10. STATISTICA 10 was also used for bar charts.

### 4.4. Cutting and Stretching Explants

The procedure for preparing explants was adapted from Belousov et al., 1999. Xenopus embryos were grown until st. 11–11.5. At this stage, the embryos were devitellinized using forceps. Then, explants were cut off in 0.8× MMR solution by using an eye micro-knife and a glass rod, combined in pairs, with inner sides to each other, and head to head (that is, dorsal side to dorsal), pinned to an agarose substrate by thin glass needles, and stretched within 3 h (see Supplementary Figure S4 for details). Control explants were pinned to agarose, but not stretched. After 3 h, stretched and control explants were lysed in TRIzol for further RNA isolation and analysis.

### 4.5. High-Throughput Sequencing

All RNA purifications and RNA-seq analysis were performed using Genoanalytica (Moscow, Russia). Briefly, total RNA was extracted from the samples with Trizol reagent according to the manufacturer’s protocol. Quality of RNA was checked with BioAnalyser and RNA 6000 Nano Kit (Agilent, Santa Clara, CA, USA). Poly(A) RNA was purified using Dynabeads^®^ mRNA Purification Kit (Ambion, Naugatuck, CT, USA). Illumina library was made from poly(A) NEBNext^®^ Ultra™ II RNA Library Prep Kit for Illumina (New England Biolabs, Ipswich, MA, USA) according to the manufacturer’s protocol. Sequencing was performed on Illumina HiSeq1500 (Illumina, San Diego, CA, USA) with 50 bp read length. At least 30 million reads were generated for each sample. Reads were mapped to the genome using Star Aligner (http://code.google.com/p/rna-star/, accessed on 15 September 2013) and the fold changes of gene expression were calculated using DEseq2.0 software (https://www.encodeproject.org/software/deseq2/, accessed on 15 September 2013). Raw RNA-seq data were deposited to GEO database under the accession number GSE246027.

### 4.6. Xenopus Single-Cell Transcriptomic Data Analysis

To analyze the publicly available database “Xenopus Jamboree!” (https://kleintools.hms.harvard.edu/tools/currentDatasetsList_xenopus_v2.html, accessed on 15 September 2019) [24], we used scripts deposited under the BSD-2 license https://github.com/comcon1/xenopus-jamboree-analyzer, accessed on 15 September 2019 as described recently [75]. As a result, for each of the identified eight stretch-responsive genes (*cdx4*, *tbxt, xmc*, *cd82*, *cebpa*, *gsc*, *otx2*, and *six3*), the relative percentage of reads contained in five conditional groups, including the following cell types represented in Jamboree database for the midneurula stage embryo (stage 14), was calculated. (1) The group “anterior neural” included “anterior neural plate”; (2) the group “anterior non-neural” included “anterior placodal area”, “cement gland primordium”, “eye primordium”, and “anterior neural crest”; (3) the group “posterior neural” included “posterior neural plate”; (4) the group “posterior non-neural” included “presomitic mesoderm”, “somite”, and “tail bud”; (5) the group “other” included all other cell types.

### 4.7. QRT-PCR

Each sample of total RNA was isolated from a group of five double-stretched or nonstretched neuroectodermal explants using the RNA isolation kit (MASHEREY-NAGEL, Düren, Germany) according to the manufacturer’s protocol. A total of 20 such samples from stretched and nonstretched explants was analyzed. The concentration of the RNA was measured with a Qubit^®^ fluorometer (Invitrogen, Waltham, MA, USA), and RNA integrity was checked visually via gel electrophoresis. Then, 250 ng of total RNA that was extracted from each sample was reverse-transcribed in a 20 mL final volume by M-MLV reverse transcriptase (Promega, Madison, WI, USA) in the presence of 10 pmol of oligo-dT primer (Evrogen, Moscow, Russia), according to the manufacturer’s guidelines (Promega, Madison, WI, USA).

To compare the efficiency (E) of qPCR, we performed qPCR with four fivefold dilutions of cDNA templates. The averages of Ct data of each dilution from three replicates of qPCR with each pair of primers were used to determine the slope and to calculate the E value (E = 1/5^−slope^).

The PCR data were imported into Microsoft Excel 2016 and analyzed using the ΔΔCt method. The geometric mean of expression of two reference housekeeping genes, ornithine decarboxylase (ODC) and elongation factor 1alpa (EF-1alpha), was used for the normalization of the target genes’ expression values. For each gene, we performed 14 to 18 independent experiments (primers data given in Table S1).

## 5. Conclusions

In summary, our findings revealed the role of MTs not only in overall mesoderm specification, as suggested by [7], but also in the anterior–posterior patterning of the embryo, as we proposed earlier [6]. Furthermore, as some genes that we found regulate cell specification, they could be a part of the feedback loop that connects morphogenesis with spatially corrected differentiation of cell types within the embryo. However, further study is necessary to investigate the molecular basis of this feedback loop in depth.

## Data Availability

Data are contained within the article and Supplementary Materials.

## Supplementary Materials

The following supporting information can be downloaded at: https://www.mdpi.com/article/10.3390/ijms25020870/s1.
