# Supplementary material for: Mechanical Tensions Regulate Gene Expression in the Xenopus laevis Axial Tissues
Source: Int J Mol Sci. 2024 Jan 10;25(2):870. doi: 10.3390/ijms25020870 (PMC10815341; doi:10.3390/ijms25020870)
Supplement: Supplementary file 1 [file ijms-25-00870-s001.zip › supplementary figures and legends.pdf]

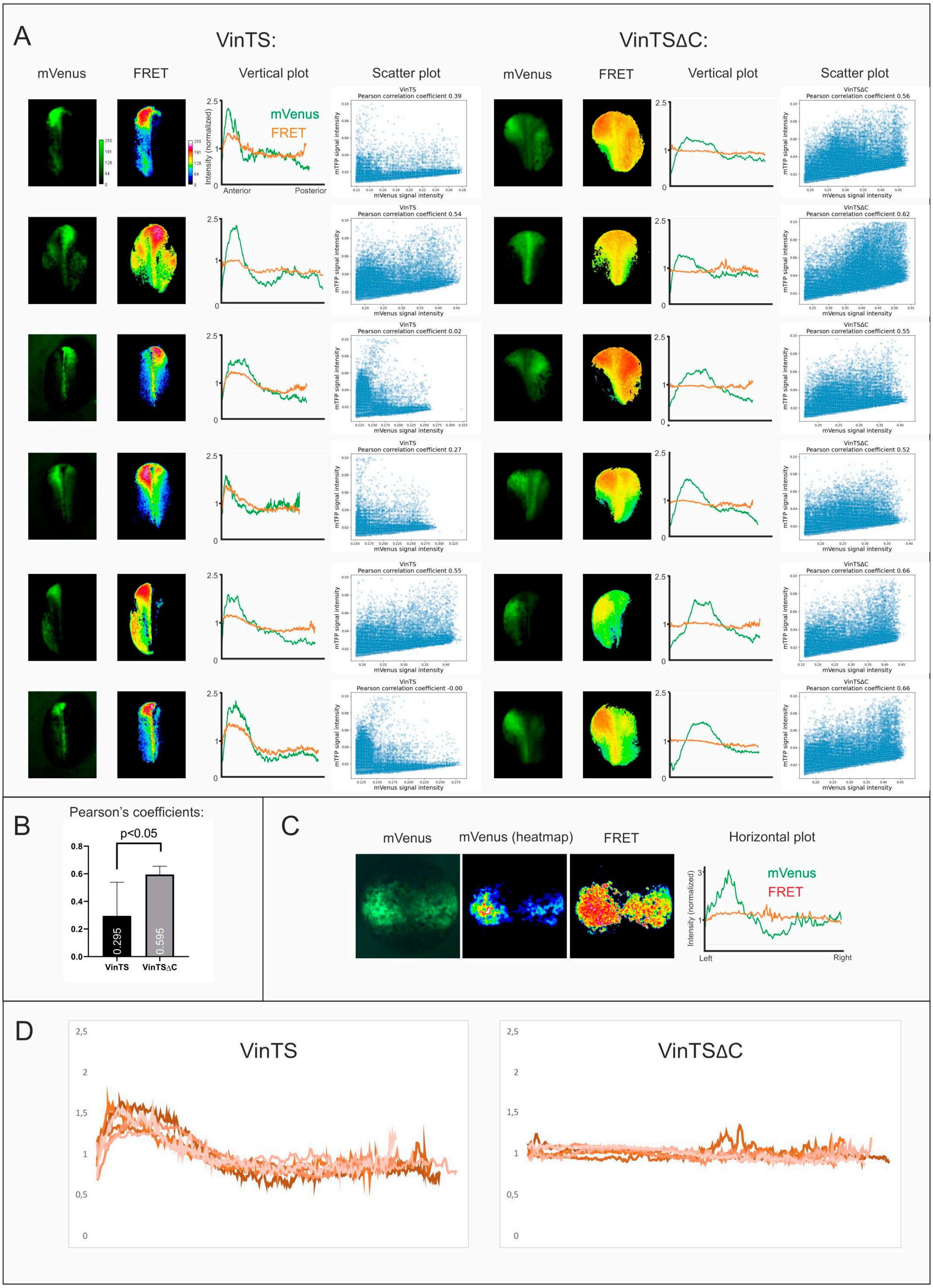

Supplementary Figure S1. Individual FRET gradients of VinTS and VinTSΔC constructs and validation of the data obtained. (A) Left panel: FRET gradients generated by VinTS construct, expressed in the neurectoderm. First row: the mVenus fluorescence showing the distribution of

VinTS in the embryo. Second row: the FRET signal. Third row: combined gradients of the mVenus fluorescence and the FRET signal along the A-P axis. Fourth row: correlations of the mVenus signal against the mTFP signal. Right panel: the corresponding data obtained for the VinTSΔC construct. (B) Comparison of the correlations of mVenus and mTFP signals of VinTS and VinTSΔC constructs given in (A). Statistical significance: Student's t-test. (C) 50pg and 25pg of *VinTS* mRNA were injected into the left and right blastomere of xenopus embryo, respectively. The resulted FRET signal was detected in the embryo's epidermis at stage 12. The horizontal left-to-right plot shows independence of the FRET signal from distribution of the VinTS construct in the embryo. (D) Summary graphs of FRET distribution along the anterior-posterior axis of VinTS and VinTSΔC constructs, respectively

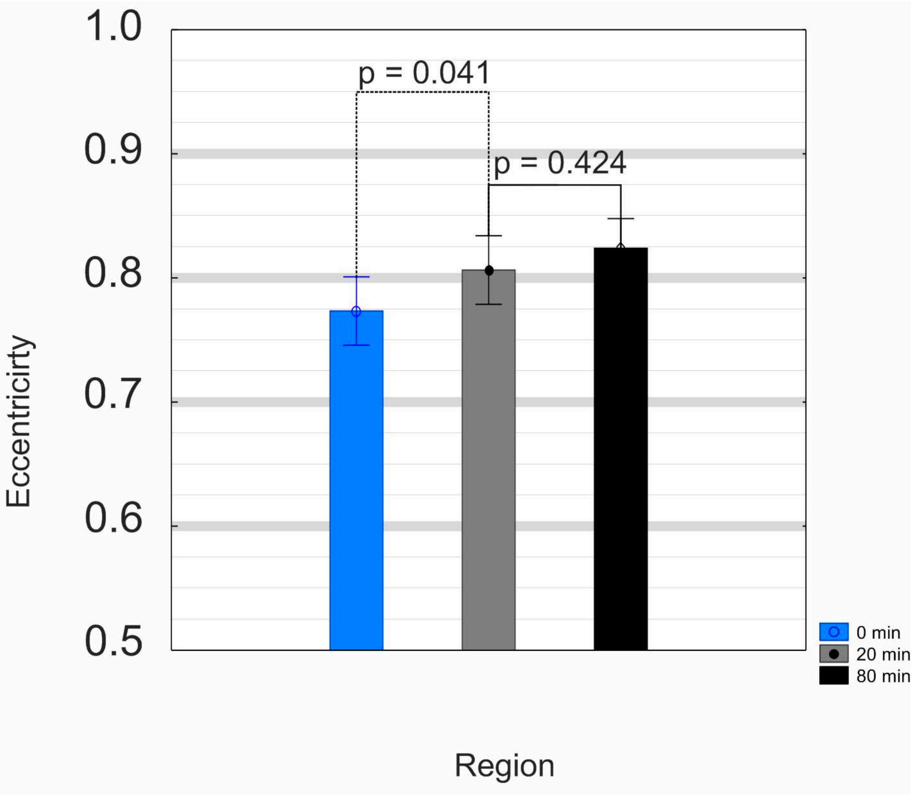

Supplementary Figure S2. To evaluate time interval sufficient for we compared mean eccentricity of cells in trunk region at 0, 20 and 80 min of observation. Note that Mann-Whitney U test shows significant difference between eccentricities of cells in trunk region at 0 and 20 min of observation (p=0,041) and not between eccentricities of cells at 20 min and 80 minutes of observation (p=0,424). Thus, we suggest 20 min interval is sufficient to detect core alteration in cell shape.

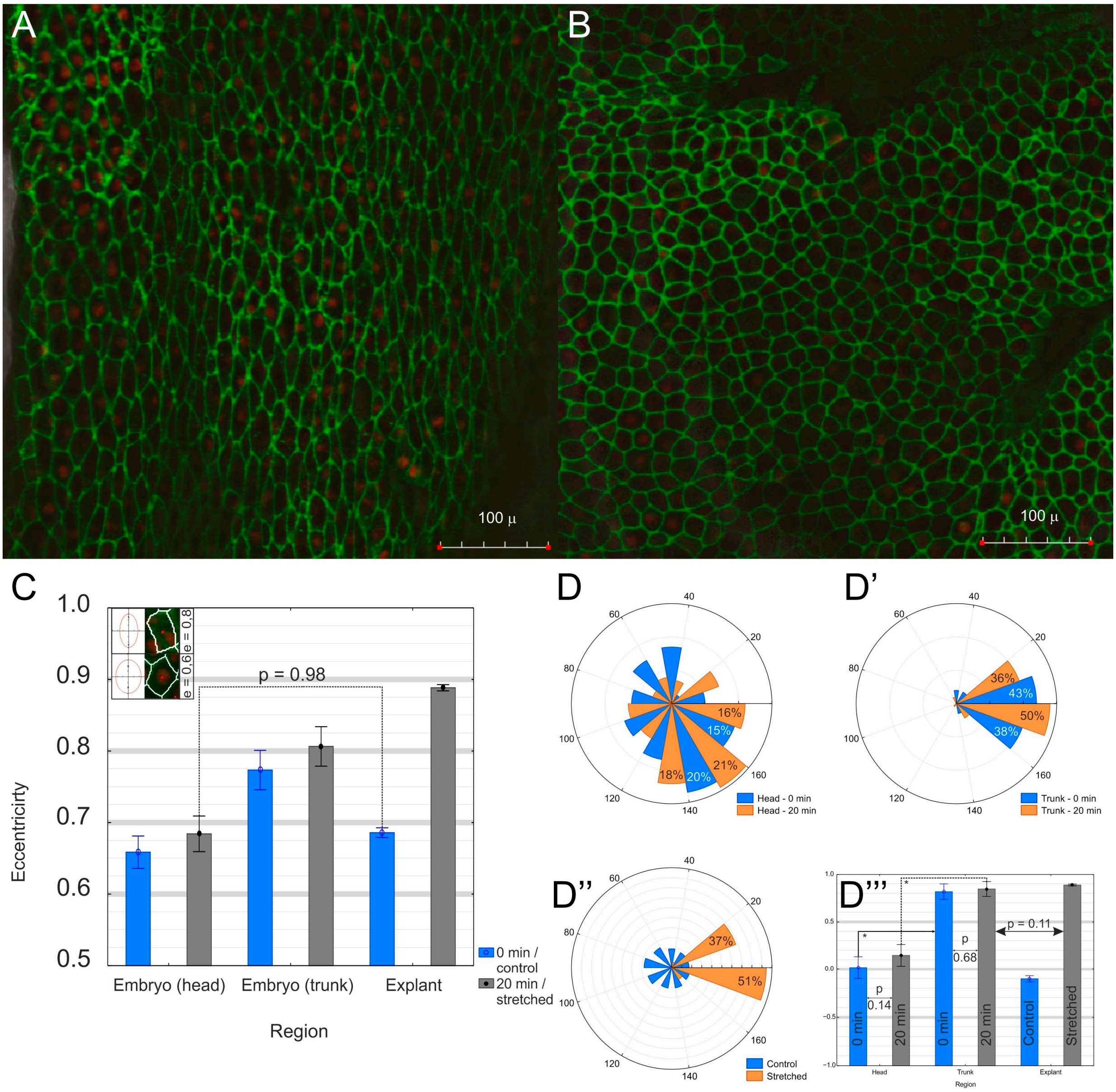

Supplementary Figure S3. Morphometric analysis of deformation in intact tissue compared to double explants. A, B: To verify, whatever stretched explants, presented in the study, are adequate representation of in vivo tissues of *X. laevis* embryo, we compared morphometric data from intact embryos with data from non-stretched (A) and stretched (B) explants (direction of stretching coincides with vertical axis). C: Eccentricity values in control explants correspond to head region at 20 min while being significantly different from both head and trunk regions at 0 min, stretched explants did not correspond to any region of intact embryos with mean value being highest among segmented samples. This may result from high magnitude of instantaneous stretching: such magnitude of deformations in normal development tends to accumulate within several hours. D: Quantification of cell orientation dynamics. D-D'': Cell's major axes orientation in intact embryos (D, D') and explants (D'''). Note that each sector corresponds to 20°-interval and can not be subdivided. Radii units denote number of observations, each tick mark equals 10 observation in case of intact embryos and 10 in case of explants. D''': Orientation dynamics quantified using order parameter. * mark statistically significant differences. Note that in control explants order parameter mean took the lowest value than in both regions of intact embryos, while in stretched explants it was indistinguishable from trunk region at 20 min.

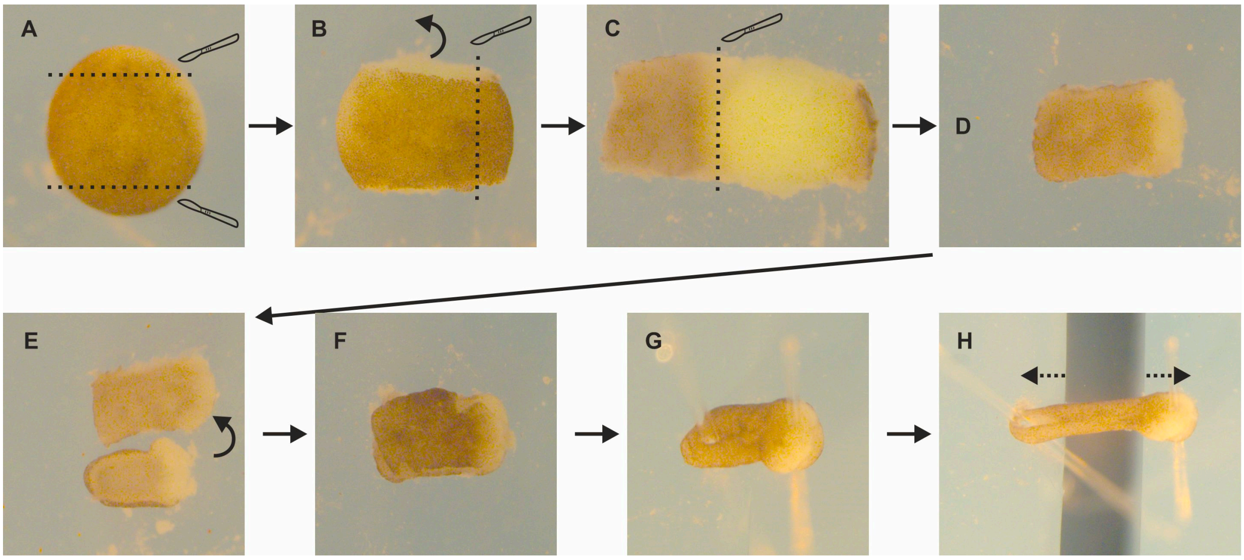

Supplementary Figure S4. Explants excision and stretching.
Dorsal side on the left in all panels.
(A) The lateral parts are cut off from the embryo. (B) The roof of the blastocoel is cut off on the ventral part and folded back. (C) The explant is prepared and cut off at the region of the dorsal blastopore lip. (D) Excised explant. (E) Two explants are stuck together in pairs with their inner sides, head to head (dorsal side to dorsal side). (F) The resulting "sandwich" is pinned (G) to agarose blocks with thin glass capillaries and (H) stretched (every 15 minutes for 3 hours). Control explants are pinned but not stretched.

Table S1. Primers for qRT-PCR.

Table S2. Genes selected as a result of transcriptomes profiling of stretched and non-stretched neurectodermal explants .

Movie S1. Video of FRET signal of VinTS mechano-reporter (Grashoff et al., 2010) [17] in the head region of Xenopus laevis embryo.

Video in green channel (left) demonstrates approximately uniform distribution of VinTS reporter in the neural anlage during gastrulation and neurulation. In the middle, video demonstrates increase of FRET intensity in the anterior neural plate of the same embryo. Schema of VinTS reporter is shown on the right.

Movie S2. Video of FRET signal of VinTSΔC (control) sensor.

Video in green channel (left) demonstrates approximately uniform distribution of VinTS**ΔC** reporter in the neural anlage during gastrulation and neurulation. In the middle, video demonstrates no significant difference of FRET intensity between the anterior and posterior parts of neural plate of the same embryo. Schema of VinTSΔC reporter is shown on the right.
