# Supplementary material for: Mechanical Tensions Regulate Gene Expression in the Xenopus laevis Axial Tissues
Source: Int J Mol Sci. 2024 Jan 10;25(2):870. doi: 10.3390/ijms25020870 (PMC10815341; doi:10.3390/ijms25020870)
Supplement: Supplementary file 1 [file ijms-25-00870-s001.zip › Table S1.pdf]

Table S1. Primers for qRT-PCR

gene	primers sequences	band length	NCBI №
Efα	GTTCATTTACCGCACAGGTTATCA<br>ACACAGGGGCATATCCAGCA	70	NM_001087442.1
ODC	GCCAGTTCTAACAAAGAAACCCA<br>TCTACGATACGATCCAGCCCA	93	NM_001086698.1
tbxt	CACCACCTACCTCAAGTCAGTA<br>TTCCATTACTTATTCCTCCAGA	95	NM_001091696.1<br>NM_001090578.1
socs3.S	CACTCGAGAGCTGTTGATTAAATGT<br>TCAGTTCTTCCGCACAGCAA	104	NM_001087305.2
mmp21.L	AAACAATCACCCCAAGGGCA<br>TTGTCGCTGACGACTTTCCA	105	NM_001085816.1
myf5	GATCTACAGACAAGTTTCT<br>AATAGGGAGGGAGCATTGCT	108	NM_001101779.2<br>XM_018256033.2
fst.L	TGGATCTTGCAACTCCATTGT<br>TCCAACAGTTTTGGGAGCTG	108	NM_001090590.1
frzb2.S	CTCTACCCGTAGAACAGTGCC<br>CAAGTATTGGCTTGGAAGTGCT	80	XM_018261409.2<br>NM_001088556.1
tbx6.S	AGTCTATACTCCTGCAGCCCC<br>TTCCGCTTGTCTTTGGCTG	117	XM_041578795.1
irx3	CTCGGTGTCTCTCACACAGCC<br>ACTGTACTGGGTCGATCTGTT	104	NM_001090735.1<br>XM_018260411.2
cer1.S	ACCTTGCCCTTCACTCAGAATA<br>TCGTCGATCTTGCTGATTTGGA	117	NM_001088331.2
adamts7.L	TGAAGGTTTCCTGTGTGGGA<br>TCTGGACAGGGGCTTCTAGT	108	XM_041586924.1
shh.L	ACTGTTCGGTCAAAGCAGAGAA<br>GGTGCCACCAAATTCCACC	92	NM_001088313.1
gbx2.1.S	GGAGAAGATGGCAATAAGACCTACA<br>CACGGACTGGACCCAGAGAA	88	NM_001090431.1
sfrp1	CCACGGTCACGAAAGACAGA<br>TCAGAGCAAAGTCACTGGCG	97	NM_001087488.1
foxc2	GGGACAGACTGCTGCACCCT<br>AAAGTCTGCTGCTGGGAGCC	90	NM_001096491.2<br>NM_001090451.1
lef1.S	GGGCCGGACCTACCTACATT<br>CATATGGTGTGGATACCAGCC	87	NM_001096734.1
nalcn.S	GATTACCAGCTGGACGGTCA<br>GTCTTGTCCACCATTTCTTCACA	105	XM_041584261.1
tbxt.2.S	CTGGTTACTCACAGTTGGGCA<br>GATGGTATTGGCAGTGGCCC	103	NM_001085559.1
gsc	AGAGAGGAAAAAGTAGAGGTTT<br>TCCACTTTTGGGCATTTTCTG	100	NM_001087809.1<br>XM_018230459.2
cd82	GCCGTGTACAAAGCGGGCT<br>ACTCCCACGCAGACTCCCA	87	NM_001092348.1<br>NM_001091678.1
prss8l.4.S	ACTTGGGCCTCCTTGGATTT<br>TGAAGGTGGGTTTGTGCTGG	109	XM_041577978.1
otx2	TGGGGGTATGGACTGTGGAT<br>CTGGTTAAGGTGACTTGTCACT	117	NM_001090691.1<br>NM_001091486.1
LOC108716930<br>(tmem116)	ATTACTGCTGACCAACGGAA<br>CACCCCATCAGGACCAGAA	119	XM_018263523.2
apobec2.L	CCGCTGCACTTACATTTGGA<br>CGCTGTAATGTCAACAGCCC	113	NM_001092842.1
tnfrsf12a.L	CCGCAGGAAAAGCAAGTTCAC<br>GTCCTATAGCTGTGACCTCTCG	102	NM_001090171.1
fbn3	AACTGCTTCCTGCAGTGTG<br>GGATGGATACAGCATTGTGT	107	XM_041579837.1<br>XM_041579835.1
LOC108700343<br>(DDB_G0277449)	GCCCGGACTCAACTCATCA<br>GACACATAACTCACCTGTCCAA	100	XM_018233450
foxc1.S	TCCTCCTCATCCAAAAATGTCC<br>TTCCGAGTTTAACACTGTCCCA	93	NM_001088214.2

six3	GCCGCTAAGAACAGGCTTCA<br>CGTCGAAGGAGACTCGGC	105	XM_018265033.2<br>NM_001085702.1
otx1.S	AACTGACCTCACTGACCCCG<br>TAACGGGTCTTGGCGAACAG	95	XM_041564173.1
aplnr.S	GCTTCTTATCCTTATGTACTGTGCC<br>TGTTAAGGCTCCCCCTCTCT	117	NM_001086378.1
frmpd1.L	CACAAACATCTCCAGGTTCAAA<br>TTTTGAGTTTCTCTGGTGCCT	102	XM_041562636
cebpa	TGTTCTGGAAAATAAAAGAAATGCC<br>AAAACAAGGATATGCGGTTGT	87	NM_001086806<br>NM_001091687
col9a1.L	GCCAAGGACCTGCTGGTAG<br>GATTCAGGCCGACGTAGACT	112	NM_001093327.1
LOC121394313	GGATGCGGCTTTCCTGAGAA<br>GGTGTAATGGAGGGGAGTGG	120	XR_005961647.1
angptl4.L	GAAGGAGAAAAGTCCAGCGGT<br>AACAGTCCACCCTGCATCTGA	99	XM_018255009
xmc	GGATACCCTTTCCCCAACTGA<br>TGGACAATTCTGCCTCCAGC	120	NM_001088686.1
chst3.L	CCTAAGGCAGCAGTGTGTGG<br>CACACGGTCTGTGTTTATCTTGT	98	XM_018225493
neurog2.L	GACTCGACCCAACTCACCTG<br>TGTCGCAAGTGACAATGCTG	94	NM_001088333
serpinh1.S	CAGCCATGATCTCCAGAAACA<br>TGGCCAGGTAGAGGTCCTTC	110	NM_001086790
bmpr1b.L	CGATGCATATCGGGAGGTATACTA<br>TGTGGGCCGTAATCTCCTG	120	XM_041590765
stx1a.L	CACCTACAGAAGAACAGGGTGA<br>TGACTGCCTTCTTGGTGTCA	105	XM_018246184
nek5.L	GCTCCGAGACTTGTAGAAGGG<br>AACTTTACTCCACCCTCAGC	110	XM_018247613
cdx4	AAGACTCTCTGAAAGACAGGTAAA<br>CTCTGCACAGAACACATGCC	111	NM_001087251<br>XM_018232950
ankfn1l.L	ATCATCTCATTTGGACTACTTCCCA<br>CGGAGCAGGAATGAGATCTGT	85	XM_041577568
ccn1l.L	CTGCTGCCTGTCATTGGGAT<br>GCAGGAGGCTTCCATCAGTT	108	NM_001091541
gypc.L	TGCTTGTGGATCTGCCTGAC<br>CATTCTCTGCAGGTGGCGTT	116	XM_041576187.1
itga2.L	AGCCTGGAGAGAAATCGGAA<br>GAAGAAGCCGAGCTTCCACA	110	XM_018266172.2
cdx1.L	CAGACATCCAGCGCCTTG<br>ACCAGATTTTTACCTGCCGT	120	XM_018251931.2
kctd12.L	AGCACTGGAATGGACAACCT<br>GCTCCCAGAATTCCCCAACA	97	NM_001094975
hes3.L	AGAGAAAGCTGGAGAAAGCAGAT<br>TGTACAGTTGAGTTCCGTGAATA	90	XM_018226118.2
gpx3.S	AGATGGGGCTCAAACTTAGGG<br>AGTTGTAGCAATCCACCGATTTT	105	NM_001092673
snai2.L	CGCACTGAAAATGCCACGA<br>ATGGGGAGATGATCACTGTATGG	104	NM_001086282
helq.S	GTCAAACTGTAAAAACTAGTGCAGA<br>GAATTTTGCAGACACATGCCA	113	XM_041579752
chad.L	CCACCCTACAGAGCCTAGCA<br>CTGCCATCTATCCACCTCCG	85	XM_018235299.2
ppp1r9b.L	CGCTACAAGAGAAGGTTGCAG<br>ACAAGCAGTAAGCACCACCA	120	XM_018234825.2
neurog1.L	CACACGAGCTCATTGCACAG<br>TCCATGTTGGAAGCCATTATCA	113	XM_018252019.2
c2orf50.L	CCGAGTGCCAAGGAGAAGG<br>TCCCCAATTCTCATGCCAGC	120	XM_018264012.2
LOC108701132	AGCAGCAAGAGACCGAGATG<br>CTGGATTGCACTTGATGCCG	99	XM_018235361.2

LOC108696684 (interleukin-11)	AATCATACCACAACTTTAAAGCCA<br>ACAGCATTCATTGAGACTACAGG	85	XM_018226270.2
tshb.S	CCCTGCCCAAAAGAGAACCA<br>AAACACACACGAAAGCAGGC	91	XM_018249167.2
LOC108699267 (neuropeptide Y receptor type 2)	CGGAAGGCAACATCCCATAAG<br>GCTTCAGACATCCTTTGCACC	112	XM_018231250.2
tbx6r.L	GTGTGACGGGCTACAGGTTT<br>GAAGTTGGGCCCCAGAGAG	101	NM_001097074
LOC121398071 (histone H2A type 1-like)	TCACTATCGCTCAGGGTGGA<br>AGCTCACTTGCTCTTAGCCG	95	XM_041576621.1
LOC108702520 (Hox-B8)	ATGAGACCACAAGCCGCC<br>TTTCGGGTCAGGTAGGGGTT	107	XM_018238034.2
ddit3.L	CCAGTTGTTTCAGAGCAGGAGA<br>TGTCCACGAGCAAAGGAGAC	91	NM_001089166
acsbg2.S	CTCTCAAAGAGCAGGTTCCA<br>TGTCTGCATTCACATTGCACTTA	105	XM_041579918
jam3.S	AGGACAAATGATGAGGGCGA<br>GTTAAGAGAGGAGTGGCGGG	100	XM_018227682.2
fstl1.L	GAGCTGAGACCCAAGTCCAA<br>ATCCCCCTCCTTCCCTTCAC	92	NM_001095580
hgf.S	GAACTACAAGTGCAAGGATCTGA<br>TTATCTGTGAGCAGTGTCCAATACG	116	XM_018255753.2
rgs20.L	GGATGGCGTGTGAAGACCTC<br>GGAGTCCAAGCTGACCTCTTT	119	NM_001093411
slc25a12.L	AGACCACCTACACTGGGGTT<br>GCGAAACACTCGAGCTCCT	98	NM_001091799
gcnt1.L	TTTGGCTGTCATTGTGGTCC<br>TGACGAATGGCACGAAGTGA	115	NM_001087338
