# Supplementary figures and images for: Mechanical Tensions Regulate Gene Expression in the Xenopus laevis Axial Tissues
Source: Int J Mol Sci. 2024 Jan 10;25(2):870. doi: 10.3390/ijms25020870 (PMC10815341; doi:10.3390/ijms25020870)

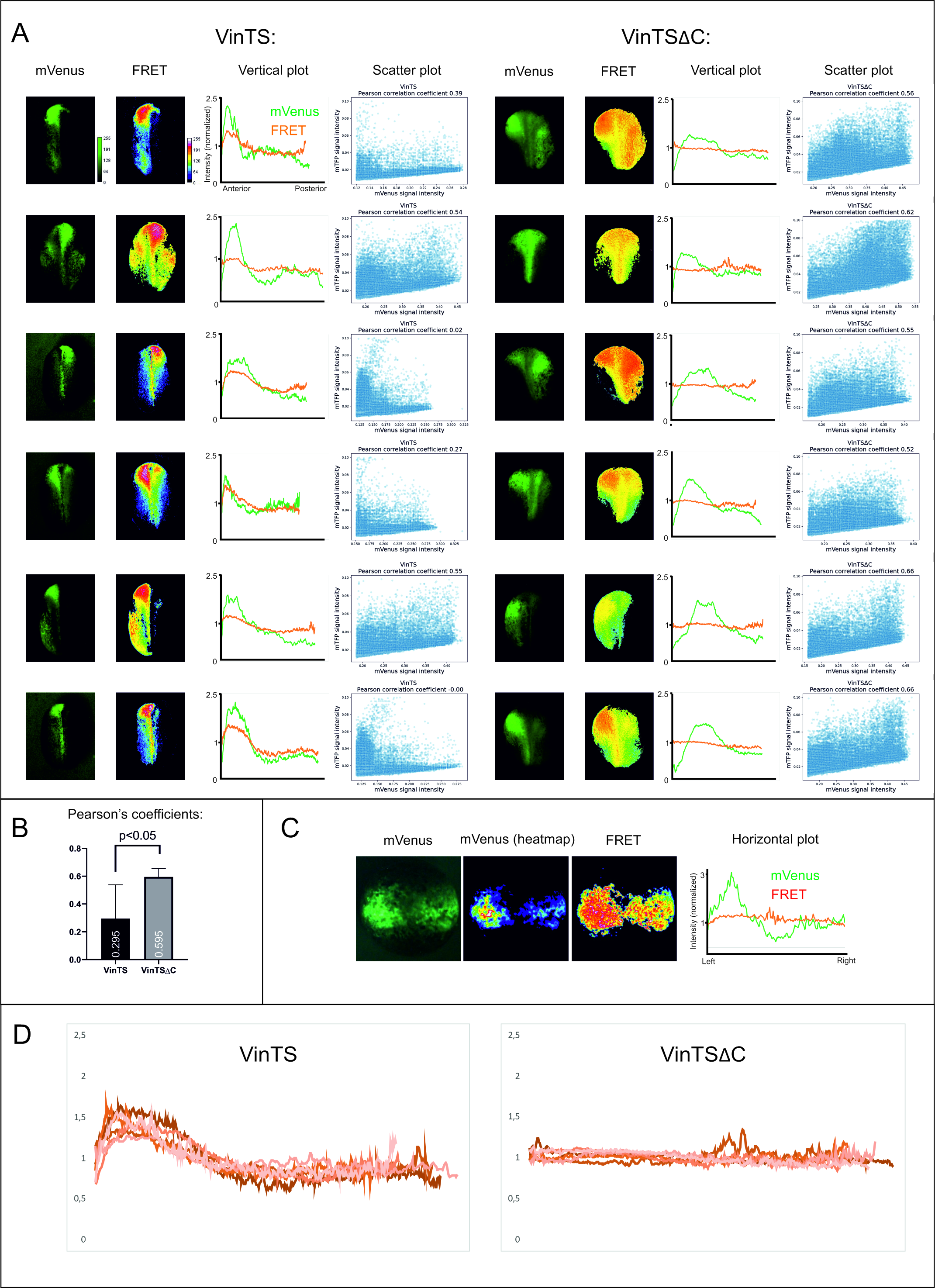

Supplement: Supplementary file 1 [file ijms-25-00870-s001.zip › Fig. S1.tif]

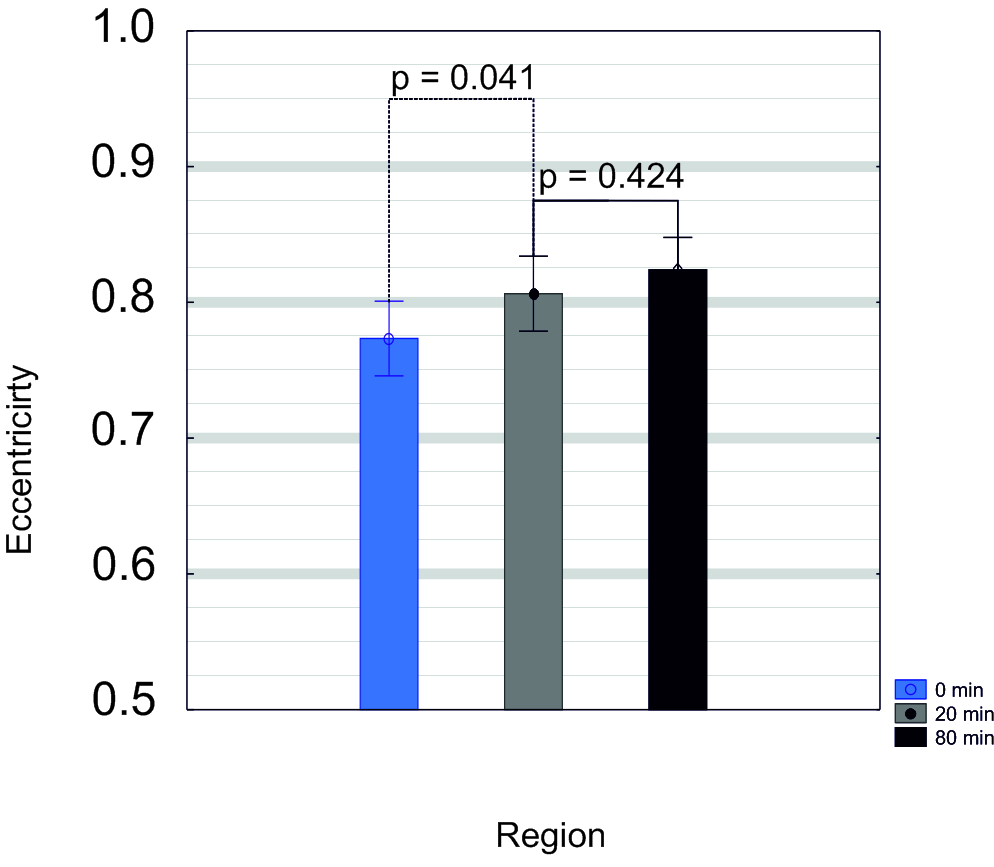

Supplement: Supplementary file 1 [file ijms-25-00870-s001.zip › Fig. S2.tif]

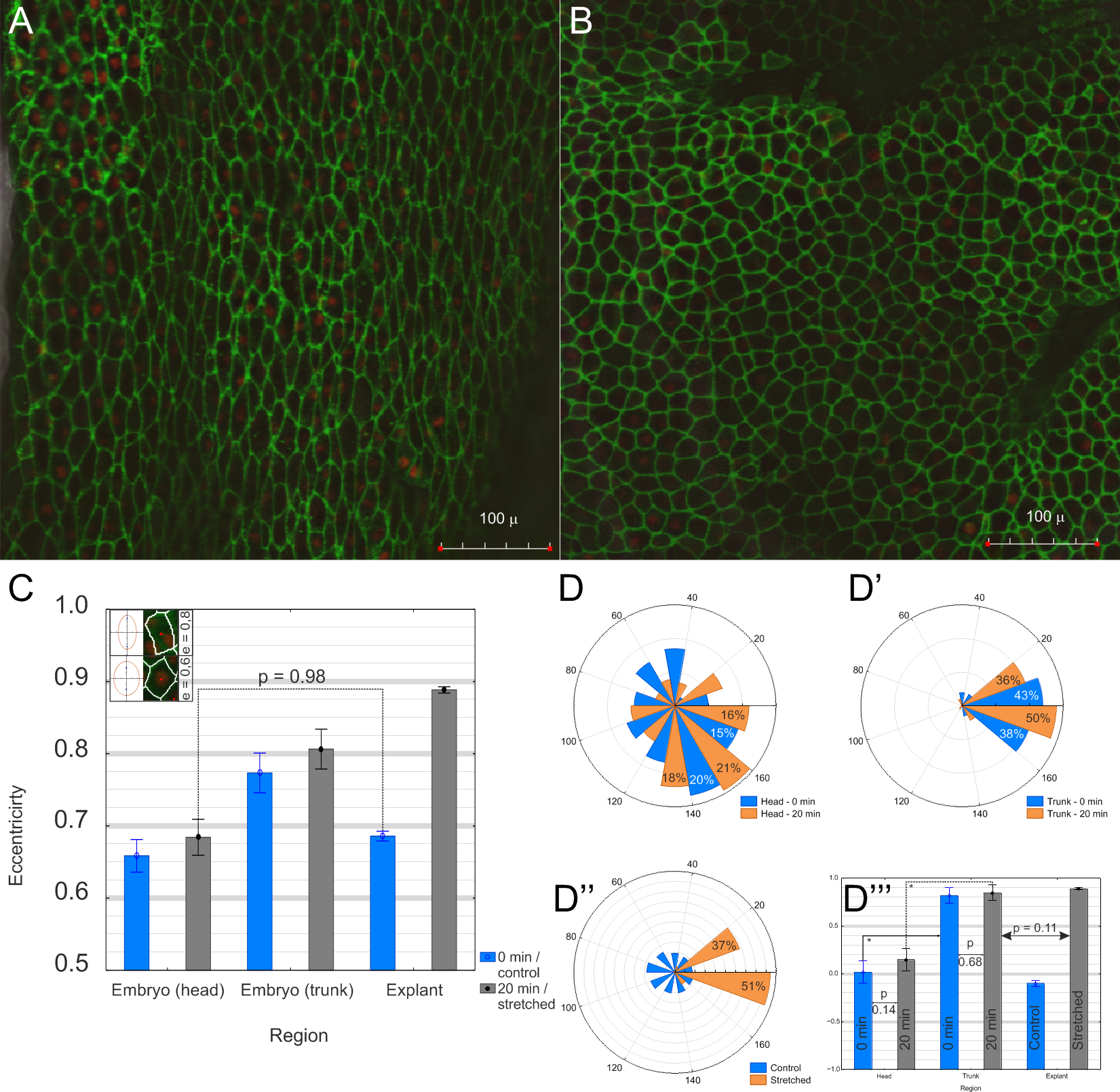

Supplement: Supplementary file 1 [file ijms-25-00870-s001.zip › Fig. S3.tif]

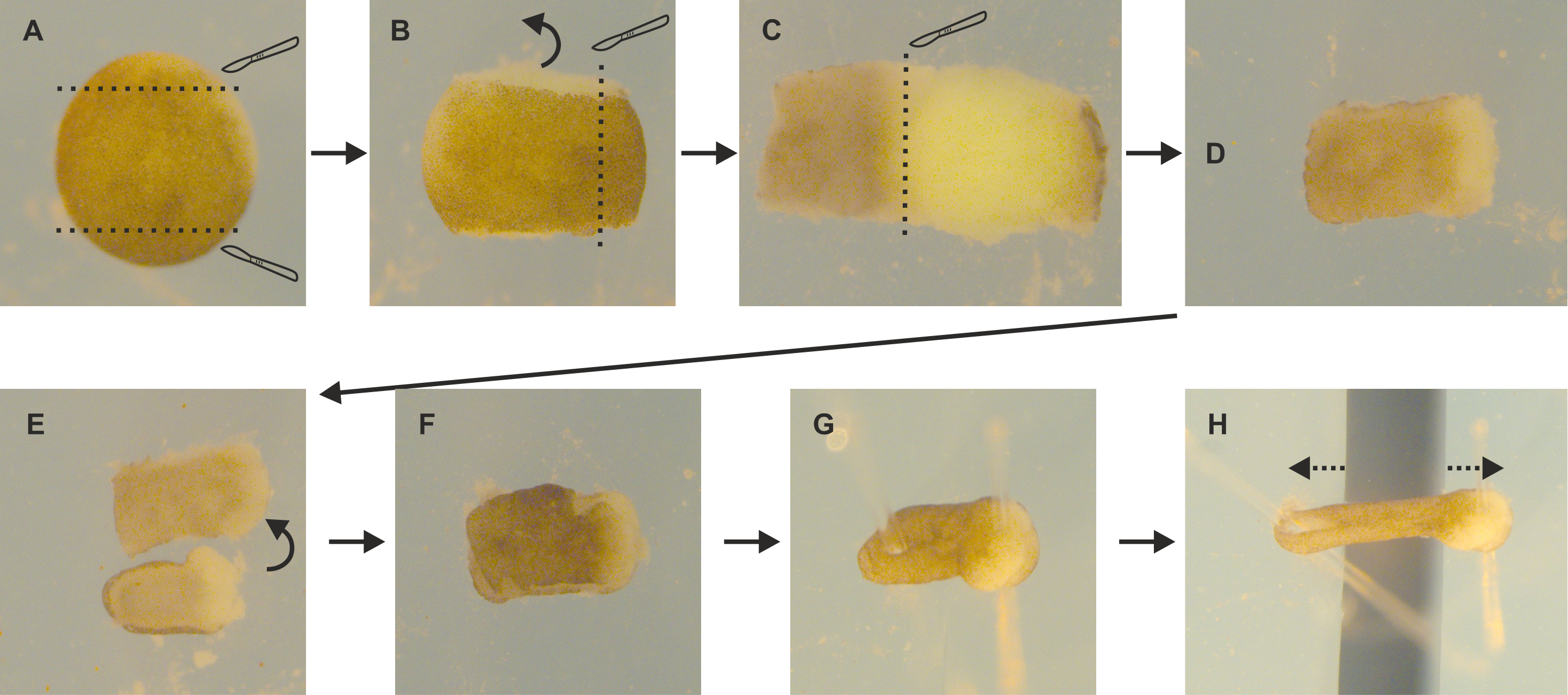

Supplement: Supplementary file 1 [file ijms-25-00870-s001.zip › Fig. S4.tif]
